# Editorial: The emerging role of biomarker mutations in early stage non-small cell lung cancer (NSCLC) management

**DOI:** 10.3389/fsurg.2026.1978192

**Published:** 2026-09-30

**Authors:** Riya Aggarwal, Adrian Valderrama, Shreya Guha, Ivana Vasic, Jeffrey B. Velotta

**Affiliations:** 1 Michigan State University College of Human Medicine, Grand Rapids, MI, United States; 2 Department of Surgery, UCSF East Bay, Oakland, CA, United States; 3 Department of Thoracic Surgery, Kaiser Permanente Oakland Medical Center, Oakland, CA, United States; 4 California Northstate University College of Medicine, Elk Grove, CA, United States; 5 Kaiser Permanente Bernard J. Tyson School of Medicine, Pasadena, CA, United States

**Keywords:** biomarkers, molecular testing, non small cell lunc cancer, personalized medicine, targeted immunotherapy

## Introduction

Precision oncology has reshaped the management of resectable non-small cell lung cancer (NSCLC). Once used in treatment in metastatic disease, biomarker testing has increasingly informed therapeutic selection earlier in the disease course, as demonstrated by the perioperative incorporation of molecularly targeted and immunotherapeutic strategies. In early-stage NSCLC, biomarkers extend beyond oncogenic drivers and PD-L1 to include systemic inflammatory measures, epigenetic regulators, and patient-level characteristics.

This editorial examines biomarkers in NSCLC through a clinical and translational perspective (Table 1). We highlight radiographic prediction of EGFR mutation status in ground-glass nodules and discrimination between common EGFR variants using CT-based models (Li et al.; Han et al.); real-world patterns and inequities in biomarker testing (Carroway et al.); the prognostic value of preoperative absolute neutrophil count (ANC) (Wang et al.); and the therapeutic relevance of the m6A regulatory protein KIAA1429 (Liu et al.). This editorial also illustrates the complexity of biomarker-guided management (Ma et al.), while placing precision care in the broader epidemiologic context of the increasing burden of lung adenocarcinoma among never-smokers (1).

**Table 1:** Characteristics of included studies.

Study	Design	N	Key finding
Carroway et al. (2025)	Retrospective cohort	8,267	38.9% overall biomarker testing rate (6.9% in stage I); NGS testing independently associated with 13% lower 3-year mortality
Han et al. (2025)	Retrospective, model comparison	193	Logistic regression (AUC 0.740) and decision tree (AUC 0.712) models both identify age and brain metastasis as key predictors of EGFR 19Del vs. 21L858R
Li et al. (2025)	Retrospective, nomogram development/validation	935	CT-based nomogram (diameter, CTR, mean attenuation, air bronchogram, vascular convergence) predicts EGFR mutation in ground-glass nodules; AUC 0.87
Liu et al. (2025)	Mini review	–	Synthesizes KIAA1429 (m6A methyltransferase) biology driving NSCLC proliferation, invasion, and gefitinib resistance
Ma et al. (2026)	Case report	1	Rare EGFR exon 18 mutation+high PD-L1 + active RA; ICI-triggered flare managed with switch to chemo-bevacizumab, enabling conversion surgery
Virata et al. (2026)	Narrative review	–	Reviews rising lung adenocarcinoma burden in never-smoking females; implicates ER*β* signaling, EGFR enrichment, and non-tobacco exposures
Wang et al. (2025)	Retrospective cohort	301	Preoperative ANC predicts DFS/OS in CEA-normal stage I NSCLC; not independent on multivariable analysis

Collectively, these contributions emphasize that the value of biomarkers depends not only on identifying molecular alterations, but also on timely testing, appropriate interpretation, integration with imaging, and equitable access to care.

### Improving biomarker identification and molecular characterization

Before biomarkers can guide treatment selection, they must be accurately and consistently identified. Recent work highlights two needs in early-stage NSCLC: expanding equitable access to tissue-based molecular testing and developing noninvasive tools that may help prioritize patients for testing. An integrated health-system cohort of 8,267 patients with NSCLC found that only 38.9% underwent biomarker testing between 2013 and 2020 (Carroway et al.). Testing was particularly limited among patients with stage I disease (6.9%) and varied by age, race/ethnicity, smoking history, socioeconomic deprivation, histology, and stage, underscoring persistent disparities in real-world molecular characterization. Notably, next-generation sequencing was associated with lower 3-year all-cause mortality.

Imaging-based prediction models can complement, but should not replace, molecular testing. A study of 935 patients developed and validated a CT-based model for EGFR mutation status in 935 patients with ground-glass nodules, integrating nodule diameter, consolidation-to-tumor ratio, mean CT attenuation, air bronchogram, and vascular convergence, resulting in strong discrimination (Li et al.). Similarly, a retrospective study used clinical and CT features to distinguish EGFR exon 19 deletion from L858R mutations, achieving moderate discrimination (Han et al.). Together, these studies support a future in which imaging can identify patients most likely to have actionable mutations.

### Expanding the definition of clinically relevant biomarkers

Biomarkers should be understood as part of the multifactorial and complex regulatory pathways rather than a single “magic” molecule acting in isolation. Tumor progression is regulated at multiple check points including DNA replication, RNA and protein synthesis, and post translational modifications. A recent review reported evidence of upregulated KIAA1429 in NSCLC (Liu et al.). KIAA1429, also known as VIRNA, encodes a core regulatory protein subunit involved in RNA methylation. Upregulation of this gene promotes anti-apoptotic processes, increases proliferative capacities, and facilitates cell cycle progression in NSCLC cells.

Biomarker utility may also vary by disease stage. For example, carcinoembryonic antigen (CEA) is a valuable prognostic marker in advanced stage NSCLC, but its sensitivity is much lower in stage I cancers. A retrospective study evaluated preoperative ANC in patients with normal CEA in stage I NSCLC and found that higher ANC was associated with worse overall survival, although ANC was not an independent prognostic factor (Wang et al.). These findings highlight the potential value of integrating molecular and systemic biomarkers to improve risk stratification in early stage NSCLC.

### Translating biomarkers into personalized clinical management

Biomarkers derive their greatest value when integrated with clinical context rather than in isolation. A case report of a 41-year-old male patient illustrates this principle in a patient with an uncommon EGFR exon 18 mutation, high PD-L1 expression, and active rheumatoid arthritis (Ma et al.). Despite a PD-L1 tumor proportion score of 55%, immunotherapy precipitated an autoimmune flare, demonstrating that a predictive biomarker cannot be considered independently of comorbidity or driver genotype. Transition to chemotherapy plus bevacizumab produced a major response and ultimately enabled conversion surgery. Although this case involved advanced disease, it illustrates a broader principle increasingly relevant to early-stage NSCLC: molecular biomarkers must be interpreted alongside patient-specific clinical factors to select the best treatment strategy.

This concept is broadened by emphasizing population-level heterogeneity (1). Lung adenocarcinoma in women, particularly never-smokers, is enriched for EGFR alterations and may reflect interactions among hormonal signaling, molecular features, and non-tobacco exposures. Together, these studies demonstrate that precision oncology requires integration of tumor-level and patient-level information. In early-stage NSCLC, this increasingly has direct therapeutic implications: EGFR and ALK status can identify patients for adjuvant targeted therapy and inform selection among perioperative systemic strategies. The challenge is therefore shifting from biomarker discovery toward timely interpretation and incorporation of results into individualized clinical decisions.

### Future directions

Future studies should determine not only who benefits from biomarker-directed perioperative therapy, but also when treatment is needed and for how long. Current perioperative immunotherapy regimens often continue treatment after surgery, yet the independent contribution of the adjuvant phase remains uncertain, particularly for those with complete pathologic response (2, 3). Molecular and response biomarkers, including pathologic response, circulating tumor DNA, defined minimal residual disease, and dynamic changes in PD-L1 may eventually guide postoperative escalation or de-escalation. The NeoADAURA trial also demonstrates the potential to move targeted therapy into the neoadjuvant setting for EGFR-mutated disease (4). Similar strategies should be evaluated across additional actionable alterations, alongside efforts to standardize testing and ensure equitable access.

## Conclusion

The studies in this article reflect the rapidly expanding scope of precision oncology in NSCLC. Biomarkers now encompass genomic alterations, imaging features, inflammatory markers, epigenetic regulators, and patient-level characteristics, with applications spanning detection, prognostication, and treatment selection. Their full clinical value, however, will depend on reliable testing and effective translation into multidisciplinary care. As biomarker-guided therapies move earlier in the disease course, integrating these diverse signals may enable increasingly individualized treatment while minimizing unnecessary therapy and toxicity.
